# Adherence to HIV treatment guidelines for initiation of antiretroviral therapy in Australia

**DOI:** 10.1186/1758-2652-13-S4-P172

**Published:** 2010-11-08

**Authors:** M Bloch, J Hoy, N Cunningham, N Roth, N Andrianopoulos, C Rayner, A Carr

**Affiliations:** 1 Holdsworth House Medical Practice, Sydney, Australia; 2 Alfred Hospital, Monash University, Melbourne, Australia; 3 Prahan Market Clinic, Melbourne, Australia; 4 Monash University, Melbourne, Australia; 5 St Vincents Hospital, Sydney, Australia

## Background

In Australia, the DHHS guidelines for treatment of adults and adolescents have been adopted with an Australian Commentary.

## Purpose of the study

An audit was conducted to determine adherence to these guidelines.

## Methods

Case records were examined of 500 sequential adults initiating ART in primary care and hospital sites (125 per site) in Sydney and Melbourne from 2004 to 2008 inclusive. Data on ART initiation and regimen prescribed, as well as adherence to all specific guidelines on patient management and monitoring, were recorded.

## Results

Of 500 subjects initiating ART (by 54 physicians with mean 14 years HIV experience), 95% were male (mean age 40 years, CD4 count 287 cells/µL, HIV RNA 89,000 copies/mL). ART initiation was mean 3.1 years after diagnosis, via clinical trial in 20.4% and as hospital in-patient in 7.7%. For "When to start", adherence to Dec 1 2009 guidelines was 91%, 82% for Nov 3 2008 guidelines, and 88% for guidelines current at ART initiation. Preferred or alternative regimens were prescribed (after exclusion of patients receiving experimental clinical trial regimens) in 79%, 90%, and 89%, according to 2009, 2008 and guidelines current at ART initiation, respectively. Preferred regimen was prescribed according to guidelines in 50%, 55% and 65%, respectively. Contraindicated ART was prescribed in 4%. Strong recommendations (level A) in 2008 guidelines were adhered to variably: for hepatitis serology (74%), oral/dental check (63%), fasting lipids (52%), fasting glucose (47%), CMV serology (50%), resistance testing (48%), vaccination history 39%, Chlamydia screening (38%), gonorrhea screening (36%), chest X ray (35%), pap smear (32%), urinalysis (26%), and TB testing (9%). Sydney patients were more likely than Melbourne patients to have co-morbidity history assessed (96% vs 62%, p<0.0001), and to commence ART via a clinical trial (36% vs 5%, p<0.0001), but less likely to have treatment adherence ability assessed (62 vs 84%, p<0.0001) with no significant difference in STI testing. Hospital sites were more likely than GP sites to perform investigations - resistance testing (71 vs 46%, p<0.0001), but less likely to discuss lifestyle health promotion (36% vs 63%, p<0.001).

## Conclusions

HIV treatment guidelines in primary care and hospital sites in Australia have been largely adhered to for when to start and what to start with, but less closely followed for co-morbidity related parameters.

